# Exploring Cannabinoids as Potential Inhibitors of SARS-CoV-2 Papain-like Protease: Insights from Computational Analysis and Molecular Dynamics Simulations

**DOI:** 10.3390/v16060878

**Published:** 2024-05-30

**Authors:** Jamie Holmes, Shahidul M. Islam, Kimberly A. Milligan

**Affiliations:** Department of Chemistry, Delaware State University, 1200 N. DuPont Hwy, Dover, DE 19901, USA; jamieholmes@desu.edu (J.H.); kmilligan@desu.edu (K.A.M.)

**Keywords:** SARS-CoV-2, PLpro, cannabis compounds, molecular docking, molecular dynamics simulations, antiviral therapy, viral replication, computational methods

## Abstract

The emergence of the severe acute respiratory syndrome coronavirus 2 (SARS-CoV-2) has triggered a global COVID-19 pandemic, challenging healthcare systems worldwide. Effective therapeutic strategies against this novel coronavirus remain limited, underscoring the urgent need for innovative approaches. The present research investigates the potential of cannabis compounds as therapeutic agents against SARS-CoV-2 through their interaction with the virus’s papain-like protease (PLpro) protein, a crucial element in viral replication and immune evasion. Computational methods, including molecular docking and molecular dynamics (MD) simulations, were employed to screen cannabis compounds against PLpro and analyze their binding mechanisms and interaction patterns. The results showed cannabinoids with binding affinities ranging from −6.1 kcal/mol to −4.6 kcal/mol, forming interactions with PLpro. Notably, Cannabigerolic and Cannabidiolic acids exhibited strong binding contacts with critical residues in PLpro’s active region, indicating their potential as viral replication inhibitors. MD simulations revealed the dynamic behavior of cannabinoid–PLpro complexes, highlighting stable binding conformations and conformational changes over time. These findings shed light on the mechanisms underlying cannabis interaction with SARS-CoV-2 PLpro, aiding in the rational design of antiviral therapies. Future research will focus on experimental validation, optimizing binding affinity and selectivity, and preclinical assessments to develop effective treatments against COVID-19.

## 1. Introduction

COVID-19 arises from an infectious strain of severe acute respiratory syndrome coronavirus 2 (SARS-CoV-2), belonging to the beta coronavirus category [1,2,3]. The infection with SARS-CoV-2 typically manifests in severe respiratory distress, characterized by symptoms such as shortness of breath, dry cough, and fever, leading to significant morbidity and mortality [4,5]. While vaccination remains the main strategy to lessen the severity of COVID-19 health implications, Nirmatrelvir (PF-07321332) [6], the key ingredient in Pfizer’s oral medication Paxlovid, notably reduces the risk of hospitalization or mortality by 89% when administered early in the infection process. This sets it apart from other clinically evaluated SARS-CoV-2 antivirals like remdesivir and molnupiravir. Nevertheless, Paxlovid is associated with side effects, necessitates administration within the initial five days of symptom onset, and carries the risk of resistance mutations [7,8,9,10,11,12]. Additionally, various approaches such as protein minibinders [13,14], peptides [15,16,17], decoy ACE2 proteins [18,19,20,21,22,23], monoclonal antibodies [24], and nanobodies [25] have been devised to develop antiviral therapeutics for COVID-19. However, all these approaches have limitations, such as ineffectiveness in clinical trials (especially when the SARS-CoV-2 variants change), low in vivo half-life, and side effects. Therefore, the exploration of effective treatments, particularly those of natural origin with fewer adverse reactions remains the primary approach to mitigate the severity of its health consequences. Therefore, the exploration of effective treatments, particularly those of natural origin with fewer adverse effects, is considered crucial globally [26,27].

Papain-like protease (PLpro) is a key enzyme encoded by SARS-CoV-2 that plays a crucial role in viral replication and immune evasion. Targeting PLpro presents a promising strategy for the development of antiviral therapies against COVID-19. Several studies have highlighted the potential of small molecules and natural compounds as inhibitors of PLpro, opening avenues for the exploration of novel drug candidates. Given the complex interplay between PLpro and viral replication, understanding the molecular interactions between PLpro and potential inhibitors is paramount for the rational design of antiviral agents [28].

In the quest for novel therapeutic strategies against SARS-CoV-2, natural compounds have attracted significant interest due to their diverse pharmacological properties and potential as sources of new drug candidates. Among these natural compounds, cannabis-derived molecules have emerged as promising candidates for the treatment of various diseases, including viral infections. Cannabis contains a myriad of bioactive compounds, notably cannabinoids, which have demonstrated diverse biological activities, including anti-inflammatory, immunomodulatory, and antiviral effects. This wealth of bioactivity suggests that cannabis compounds may hold potential as therapeutic agents against SARS-CoV-2.

Cannabis sativa L., an annual herbaceous plant originating from central Asia, is commonly known as Indian hemp, while “marijuana” is a term of Mexican origin that now refers to the dried flowers and leaves of the cannabis plant. The Arabic term “hashish” denotes the resin gum of the plant [28,29]. Cannabinoids, the primary active metabolites found in Cannabis sativa, are a class of terpene phenolic compounds concentrated mainly in the female flowers’ trichome cavities [30,31]. Notably, Δ-9-tetrahydrocannabinol (THC) is the principal psychoactive compound, while cannabidiol (CBD) is the main non-psychotic active compound [32]. Additionally, Cannabis sativa exhibits various therapeutic properties attributed to cannabinoids, which inhibit neurodegenerative disorders, suppress breast cancer cell proliferation, and alleviate inflammation, chronic pain, multiple sclerosis, epilepsy, glaucoma, and nausea [33,34].

The interaction between the COVID-19 virus and host cells triggers robust pro-inflammatory and immune responses, resulting in a cytokine storm and the circulation of immune cells. This immune reaction is orchestrated through complex mechanisms, involving various signaling molecules, including endocannabinoids (eCBs). Within this intricate system, the human endocannabinoid system (ECS) emerges as a crucial regulator, encompassing cannabinoid receptors type 1 (CB1) and 2 (CB2). While CB1 primarily resides in the central nervous system (CNS), CB2 is notably abundant in immune cells, where it exerts anti-inflammatory effects by suppressing pro-inflammatory cytokines and promoting the production of anti-inflammatory cytokines [35]. Moreover, CB2 plays an immunomodulatory role by regulating apoptosis, cellular proliferation, and the expression of proinflammatory cytokines, while cannabinoids exhibit affinity for various other receptors such as the G protein-coupled receptor (GPR55), transient receptor potential vanilloid (TRPV) channels, Peroxisome proliferator-activated receptors (PPARs), and serotonin 1A receptors, among others [36]. Phyto-cannabinoids have also demonstrated the ability to suppress lymphocyte proliferation and inflammatory cytokine production [37,38]. Overall, the activation of the ECS appears to play a critical role in both preventing the onset and reducing the severity of COVID-19.

To our knowledge, no study has evaluated the roles of the ECS, cannabinoids, and cannabis in the progression of SARS-CoV-2 infection. Also, no epidemiological data are available on the incidence of COVID-19 in people taking medicinal or non-medicinal cannabinoids [39,40,41,42]. Pre-existing non-medicinal consumption of cannabinoids should not be encouraged during the ongoing COVID-19 pandemic due to potential respiratory complications. Continuation or discontinuation of therapeutically prescribed cannabinoids should be discussed on a case-by-case basis, with the prescribing physician considering the risk–benefit ratio of each patient [43,44,45]. Given the large-scale, worldwide consumption of cannabinoids, medicinal or not, it appears critical to improve preclinical and clinical knowledge on cannabinoids and COVID-19 [46,47,48]. To confirm an immunomodulatory effect of cannabinoids and a potential interaction with SARS-CoV-2, in vitro and in vivo experimental models are requested. From a clinical standpoint, epidemiological studies (with case-control design) and retrospective data about the consumption of cannabinoids by patients with SARS-CoV-2 infection are needed to investigate the potential influence of cannabinoids on COVID-19 disease progression and severity [49,50].

In this context, the present research aims to investigate the potential of cannabis compounds as therapeutic agents against SARS-CoV-2 by targeting PLpro. Through computational approaches, including molecular docking and molecular dynamics simulations, we aim to elucidate the binding mechanisms and interaction patterns of cannabis compounds with PLpro. By screening a library of cannabis compounds against PLpro, we seek to identify potential inhibitors that exhibit strong binding affinities and favorable interaction profiles. Furthermore, our study aims to characterize the dynamic behavior of cannabinoid–PLpro complexes, providing insights into the stability and conformational dynamics of these interactions [51,52].

## 2. Materials and Methods

### 2.1. Retrieval and Preparation of Molecular Structures

#### 2.1.1. Retrieval of Cannabinoids from PubChem

For this study, we selected four well-known cannabinoid compounds, CBG-I, Cannabidiolic acid, CBD, and Cannabigerolic acid, to evaluate their potential against SARS-CoV-2 PLpro protein (6W9C). These four compounds were downloaded in SDF format from the Chemical Compound Deep Data Source database (https://www.molinstincts.com/, accessed on 10 November 2023). They were then evaluated based on the rule of five.

#### 2.1.2. Retrieval of SARS-CoV-2 PLpro Protein Structure from Protein Data Bank (PDB)

The three-dimensional (3D) crystallographic structure of the SARS-CoV-2 non-structural PLpro protein (PDB ID: 6W9C) [53,54] was retrieved from the Protein Data Bank (www.rcsb.org, accessed on 2 November 2023), and the sequence was obtained from the UniProt database [51,52] The resolution of the viral PLpro protein is 2.70 Å with global symmetry (cyclic–C3), and the global stoichiometry is Homo 3-mer–A3. The targeted receptor was analyzed based on physicochemical properties, such as a total structure weight of 107.81 kDa, an atom count of 7371, along one unique protein chain. The amino acid sequence contains a lot of necessary information, including extinction coefficient, theoretical pI, instability index, estimated half-life, aliphatic index, GRAVY, and amino acid composition [51,52]. Further physicochemical properties were determined via the ProtParam tool (https://web.expasy.org/protparam/, accessed on 15 November 2023). In ProtParam, the FASTA format was used to check physicochemical properties. The conserved motifs, including the catalytic triad (Cys111, His272, and Asp286), were selected based on the published literature [53,54].

### 2.2. Molecular Docking

The next step after retrieving ligands and the target protein was molecular docking to find the best binding pose of the ligands with the SARS-CoV-2 PLpro protein. AutoDock Vina, an open-source docking tool utilized for molecular docking, requires SDF/MOL and PDB files of ligands and receptors, respectively. Pyrx is a graphical interface for AutoDock Vina, a popular, robust, and user-friendly software application for predicting the binding modes and affinities of ligands to the target protein [55,56]. Pyrx streamlines the process of preparing input files, establishing parameters, and viewing docking experiment outcomes [57,58,59,60].

Molecular docking was carried out using Pyrx’s Vina wizard with default algorithms. Using the command prompt, the prepared ligands were docked to the targeted receptor one by one. A total of 9 poses of each ligand were generated. All the poses were analyzed based on binding affinity and Root Mean Square Deviation (RMSD) value. The best pose with the lowest binding affinity and RMSD was chosen, and the remaining poses were eliminated. Docking scores were obtained and saved in the .CSV file format. The docking scores (Kcal/mol) were used to calculate the ligands’ binding affinity. The best poses with binding interactions after molecular docking were assessed using PyMOL (http://www.pymol.org accessed on 11 September 2023). The interactions with the lowest binding energies were found to be the most favorable interactions.

MD simulations were conducted employing the Desmond engine and utilizing the VSGB solvent model and OPLS3e force field, following a methodology described in the prior literature. Enough water molecules were used to solvate the system, and the non-bonded interactions were treated with a cut-off range of 10 Å and periodic boundary condition was fixed with Particle Mesh Ewald (PME) method. The temperature and pressure were controlled using a Nose–Hoover thermostat and Parrinello–Rahman barostat, respectively. A timestep of 50 nanoseconds was employed for the simulations. These parameters were chosen based on established protocols for MD simulations.

#### 2.2.1. Protein Structure Preparation Using AutoDock Tools

The protein structures were prepared using AutoDock Tools v 4.2 [61,62,63]. Grid-based molecular docking was utilized to enable the binding of compounds in multiple potential conformations. Prior to the docking process, Gasteiger charges and polar hydrogens were added to the protein molecule. In the docking process, a square grid box with a size of 20 Å was used (centered around the pocket). Before docking, the target protein was prepared by removing heteroatoms, including water molecules, and small molecules via PyMOL (https://pymol.org/2/, accessed 15 October 2023). Additional metal ions and unnecessary chains were deleted during structure modification, and the lowest-state penalty was selected. Tautomeric states and protonation were adjusted to a pH of 7.4. It was verified that the 3D structure did not contain any missing residues and it was saved into PDB file format. Then, it was loaded into the workspace of Pyrx and converted into a macromolecule, which converts the receptor into pdbqt file format by adding charges to the protein structure [63]. The BIOVIA Discovery Studio 2020 client software version R1 package was used for binding site predictions, interaction analyses, and molecular visualization of docked complexes [64,65].

#### 2.2.2. Ligand Pre-Processing

In this study, four well-known cannabinoid compounds—CBG-I, Cannabidiolic acid, CBD, and Cannabigerolic acid—were chosen to assess their potential against the SARS-CoV-2 PLpro protein. These compounds were obtained in SDF format from the Chemical Compound Deep Data Source database (https://www.molinstincts.com/, accessed on 10 November 2023). Subsequently, they were evaluated based on the rule of five. The ligand underwent preprocessing using AutoDock-Tools to address any structural issues such as missing atoms, incorrect bond orders, or steric clashes. Energy minimization of the ligand structure was conducted to optimize its conformation. Appropriate protonation and ionization states were assigned to the ligand at the desired pH. Multiple low-energy conformations of the ligand were generated to accommodate flexibility during docking. Gasteiger charges were added to each conformation, and rotatable bonds were adjusted. Finally, all ligands were converted into pdbqt file format for docking [65].

#### 2.2.3. Grid-Based Docking Using AutoDock Vina

The prepared 3D structure was further processed to build a grid box on the whole protein for blind docking. The X, Y, and Z coordinate dimensions for blind docking were −154.97, 180.54, and 167.23, respectively. All three dimensions of the box were 72 × 88 × 146. For docking, the receptor was saved in the pdbqt file format.

### 2.3. Binding Site Predictions and Interaction Analyses Using BIOVIA Discovery Studio

The BIOVIA Discovery Studio 2020 client software and PyMOL were used to predict binding sites, analyze interactions, and visualize docked complexes. A target site within the protein was selected for docking, typically a pocket in its three-dimensional structure. Discovery Studio’s ‘Ligand Binding Site’ module predicted the protein’s binding site using geometric and physicochemical algorithms. The ligands were docked within the pocket in various conformations, and binding affinities were assigned to each snapshot. A 20 Å square box limited the docking process. Results outlined the ligands and their binding affinities to the protein (PLpro). Visualizations in Discovery Studio highlighted residues within 5 angstroms of the predicted binding site for further analysis [64,65].

### 2.4. Evaluation Criteria for Docking Results

The scoring function and conformational sampling were utilized to explore the ligand conformational space for docking evaluation. The scoring function of the docking software computed the expected binding affinity of each ligand-protein system, where lower binding energies indicated stronger binding interactions. The RMSD value between the docked ligand conformation and the control ligand conformation was calculated to assess the accuracy of ligand binding mode prediction. The projected binding mechanism was then assessed for plausibility and consistency by examining interactions between the ligand and binding site residues, including hydrogen bonds, hydrophobic interactions, and π–π stacking interactions.

### 2.5. Validation and Verification Techniques

To validate the docking approach, redocking experiments were carried out on known ligand–protein complexes with empirically determined binding modes. The RMSD between the docked poses and experimental binding positions was calculated to determine the accuracy of the docking predictions. Cross-docking research was carried out, which involved docking different ligands into the target protein’s binding region. The docking software’s capacity to accurately anticipate the binding modes and affinities of various ligands was assessed. The projected binding mechanism was evaluated for plausibility and consistency by analyzing particular interactions between the ligand and binding site residues, such as hydrogen bonds, hydrophobic interactions, and π–π stacking interactions. To validate and verify the docking results, molecular mechanics generalized Born and surface area (MM/GBSA) analysis was run [66].

MM-GBSA analysis was conducted via the prime module of Schrodinger to evaluate the binding free energy (ΔG(bind)) of all four systems (receptor complexed with Cannabidiolic acid, Cannabigerolic acid, CBD, and CBG compounds), which is a measure of the stability of the complex and thus the strength of the binding [67]. Counter ions were stripped, and the VSGB solvent model with OPLS3 force field was employed, along with rotamer search techniques to calculate ΔG (bind) [68,69]. The total binding free energy is the difference between the energy of the protein–ligand complex and the free energy of the individual protein and inhibitor. A stronger relationship is indicated by more negative ΔG (bind) values.

### 2.6. Molecular Dynamic Simulation

The protein–ligand complex of PLpro-CBGA underwent simulation using the Schrödinger software Maestro version 2023-1 package for a total of 50 nanoseconds. The complex was suspended in a water solvent and neutralized with charged ions. Energy minimization was conducted to relax the structure and eliminate steric hindrances. Subsequently, the solvent and ions were stabilized by adjusting temperature to 300K and pressure to 1 bar before the production step.

Schrödinger, a computational biophysics-based platform, facilitates drug discovery by characterizing protein–drug interactions via simulations. Initial parameters such as ensemble, total molecules, water molecules, simulation time, and charge are determined. Molecular docking provides the protein–ligand complex structure, which Schrödinger further refines through energy minimization and equilibration steps, ensuring favorable geometry and stability.

## 3. Results

### 3.1. Physicochemical Analysis of PLpro and Chemical Compounds

All selected compounds were evaluated based on their molecular properties, including molecular weight, hydrogen bond donors and acceptors, XLogP, rotatable bonds, heavy atoms, and polar surface area (Table 1). The standard criteria for selection were: molecular weight < 500, LogP < 5.6, H-bond donors < 5, H-bond acceptors < 10, PSA < 140, RB < 10.

The amino acid sequences of target proteins from SARS-CoV-2 were retrieved from the UniProt database, while the 3D structure was obtained from the PDB database. The theoretical pI of the protein is 7.99, with an instability index of 36.41, indicating that the targeted receptor is stable. The total number of negatively charged residues is 29, and the total number of positively charged residues is 31. The atomic composition is as follows: C (1598), H (2459), N (415), O (480), S (19). The estimated half-life is >10 h in *Escherichia coli*, with an aliphatic index of 71.07 and a grand average of hydropathicity (GRAVY) of −0.361.

### 3.2. Computational Docking of Cannabinoids with SARS-CoV-2 PLpro

To gain a better understanding of the binding energies and interactions of the selected target protein PLpro, molecular docking calculations were performed for the four selected compounds. The compounds with their docking scores and various interactions that stabilize the binding are given in Table 2. The docking scores for Cannabidiolic acid, Cannabigerolic acid, CBD, and CBG with the PLpro protein of SARS-CoV-2 are −6.1, −5.3, −5.0, and −4.6, respectively. The SARS-CoV-2 PLpro’s active site showcases a conventional catalytic triad, consisting of Cys112–His273–Asp287. Although none of our molecules bind very close to the active site, the binding of the molecules has shown increased structural dynamics which could impact the active site and consequently the function of PLpro.

### 3.3. Binding Affinity and Interaction Patterns of Cannabinoids

#### 3.3.1. Cannabigerolic Acid (CBGA)

**Figure 1:**
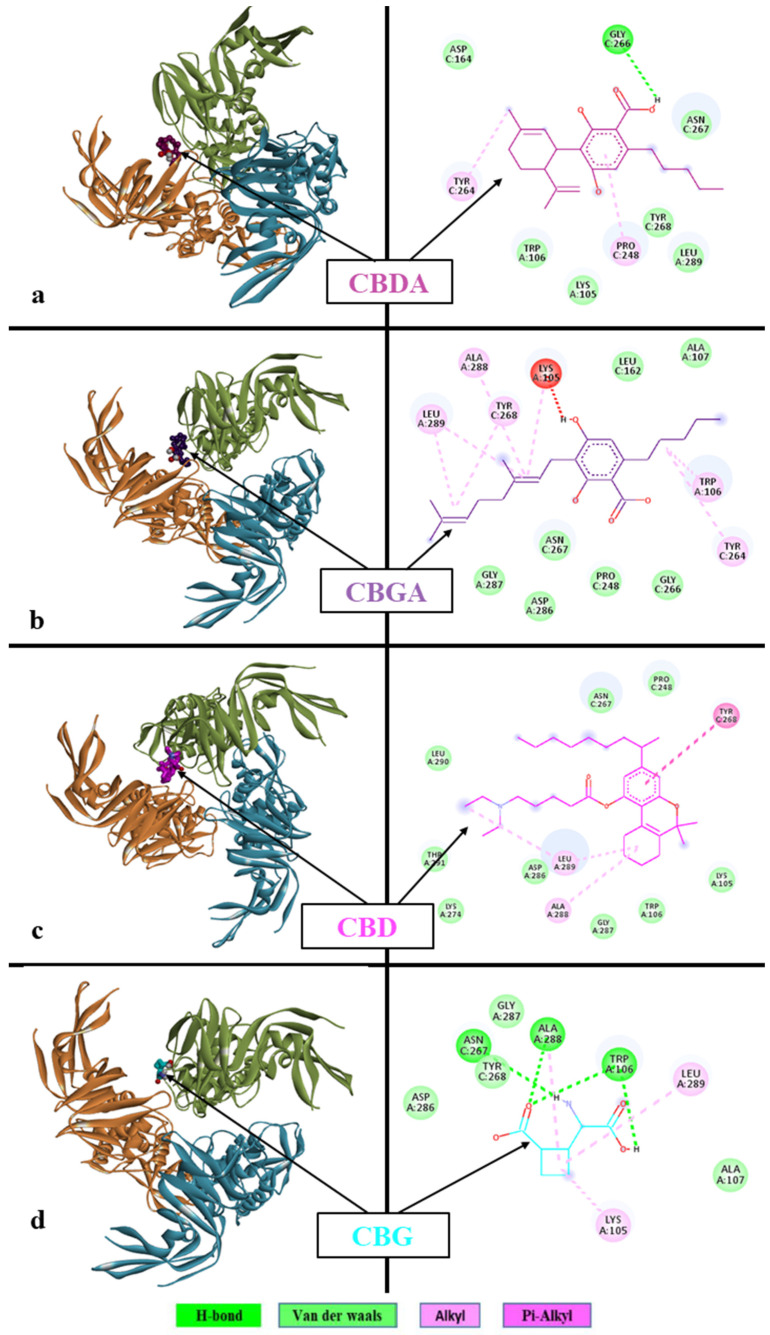
Molecular docking interaction of (**a**) CBDA, (**b**) CBGA, (**c**) CBD, and (**d**) CBG with SARS-CoV-2 PLpro protein. Ribbon diagram with the solvent surface rendered view and 2-dimensional interaction diagram showing interactions of respective cannabinoids with SARS-CoV-2, PLpro protein active site. The various interaction types are indicated by different colors provided in the color panel at the bottom. BIOVIA Discovery Studio 2020 client was used to visualize and analyze the interactions.

The docking interaction of CBGA was found to be the highest among the four cannabinoids (Figure 1).

The binding affinity of CBGA with the PLpro active site was −6.1 kcal/mol. The interaction involved the formation of eight alkyl and pi–alkyl bonds with LYS105, TRP106, TYR264, TYR268, ALA288, and LEU289.

The significance of these eight alkyl and pi–alkyl bonds to CBGA having the highest binding affinity and the interacting residues are as follows:

The eight alkyl and pi–alkyl bonds collectively contributed to the binding affinity score of −6.1 kcal/mol within the protein–ligand complex. Based on our interpretation and research, there is no particular relevance of the type of bonds, but we are rather interested in the estimated binding affinity of the protein and ligand complex.

On the protein side, the residues that participated in the bond formation were Lysine at position 105, Tryptophan at position 106, Tyrosine at position 264, Tyrosine at position 268, Alanine at position 288, and Leucine at position 289. The position here indicates the position in terms of the protein sequence.

In terms of the chemical nature of these residues: Lysine contains a positively charged side chain, and the others (Tryptophan, Tyrosine, Alanine, and Leucine) contain hydrophobic side chains.

#### 3.3.2. Cannabidiolic Acid (CBDA)

CBD docked to the SARS-CoV-2 PLpro protein demonstrated a binding affinity of −5.0 kcal/mol. The analysis showed the formation of various alkyl and conventional hydrogen bonds with nearby residues of the active site, such as PRO248, TYR264, and GLY266.

#### 3.3.3. Cannabigerol (CBG)

Cannabidiolic acid docked to the SARS-CoV-2 PLpro protein demonstrated a binding affinity of −5.3 kcal/mol. The analysis showed the formation of different pi–alkyl, alkyl, and conventional hydrogen bonds with nearby residues of the active site, such as TYR268, ALA288, and LEU289.

#### 3.3.4. CBD

CBD docked to the SARS-CoV-2 PLpro protein demonstrated a binding affinity of 4.6 kcal/mol. The analysis showed the formation of different alkyl and conventional hydrogen bonds with nearby residues of the active site such as LYS105, TRP106, ASN267, ALA288, and LEU289.

Each row indicates the docking results for a single protein–ligand complex. The left columns depict the structural overview of the interactions, where the trimers of the protein are colored in three ribbon-like structures (yellow, blue, and green). The ligand is shown in a solid structure placed in the protein pocket. The right column depicts the specific protein–ligand contacts noted during the docking process. Here, the ligand is depicted in a wire-like structure, and the amino acids from proteins are depicted in a ball-like structure. The names of the amino acids are indicated in standard three-letter notations (for example, LYS A:105 represents the Lysine residue at position 105 from the “A” chain of the protein). The dotted arrows between the ligand and amino acid indicate the observed contacts, and they are colored according to their nature (hydrogen, Van der Waals, alkyl, and pi–alkyl bonds).

#### 3.3.5. Cannabinoids with Other Targets

To gain a better understanding of the binding energies and interactions of the selected target protein 6W9C, a molecular docking process was performed for the four selected compounds. The docking scores for Cannabidiolic acid, Cannabigerolic acid, CBD, and CBG with the PLpro protein of SARS-CoV-2 are −7.1, −7.2, −7, and −5.4, respectively. The docking results were then analyzed using a cutoff value of −7, and three of the compounds’ docking scores lie within this criterion. Compounds with their docking scores are given in Table 3.

**Figure 2:**
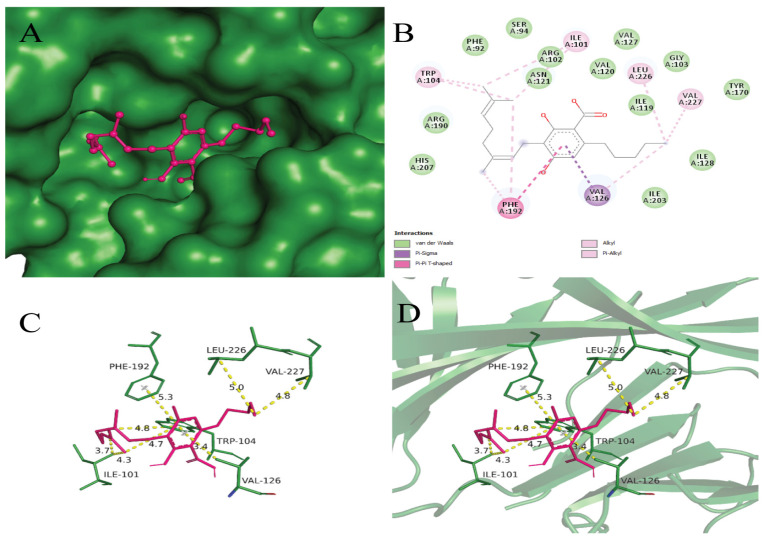
(**A**) Surface mapping of 6W9C-Cannabigerolic acid complex with forest green and hot pink colors respectively. (**B**) Showing 2D interaction view of Cannabigerolic acid with the SARS-CoV-2 PLpro receptor. The legend for the interactions involved is given in the left lower box. (**C**) Labeled interacting residues with Cannabigerolic acid and calculated distances. (**D**) Cannabigerolic acid (hot pink) and interacting residues (forest green) in stick representation, while receptor is in cartoon form with 50% transparency.

CBGA demonstrated the best binding affinity of −7.2 compared to the others. Based on the binding scores, CBGA exhibits the potential to bind and maintain stable interactions with the SARS-CoV-2 PLpro protein for an extended duration. This characteristic suggests its capacity to inhibit the virus by hindering PLpro receptor binding, thereby preventing its entry into the host body. The docking poses of CBGA with the targeted receptor were analyzed with 2D and 3D interactions along with surface mapping, as displayed in Figure 2. It was observed that CBGA makes Pi–sigma, pi–pi T-shaped, alkyl, and pi–alkyl interactions with nonpolar aliphatic residues, such as VAL126, ILE101, VAL227, LEU226, and aromatic residues PHE192 and TRP104, respectively. VAL126 depicts a 3.4 Å distance and interaction with aromatic carbon, while Ile101 depicts a 3.7 Å distance between CBGA and the target protein.

**Figure 3:**
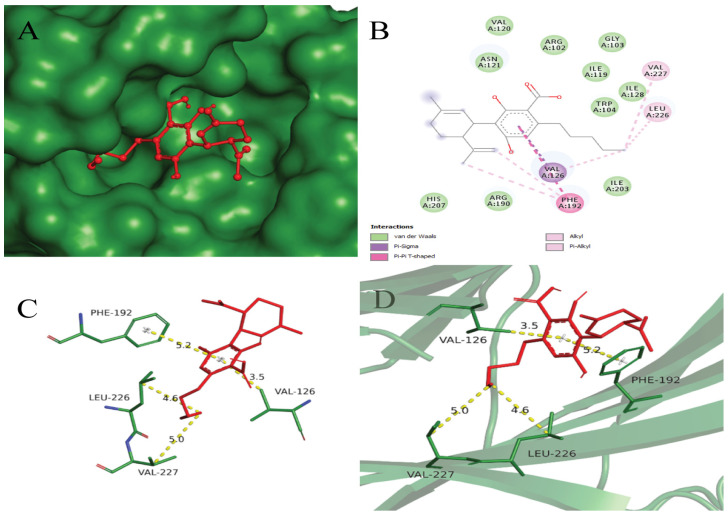
(**A**) Surface mapping of 6W9C-Cannabidiolic acid complex with forest green and red colors, respectively. (**B**) Showing 2D interaction view of Cannabidiolic acid with the SARS-CoV-2 PLpro receptor. The legend for the interactions involved is given in the left lower box. (**C**) Labeled interacting residues with Cannabidiolic acid and calculated distance. (**D**) Cannabidiolic acid (red color) and interacting residues (forest green) in stick representation, while receptor is in cartoon form with 50% transparency.

Aromatic residue PHE192 was observed to participate in pi-pi T-shaped bonding. Pi–sigma interactions were detected between the nonpolar aliphatic residue LEU226 and Cannabidiolic acid, while VAL227 and VAL126 residues were found to be involved in alkyl and pi–alkyl interactions with a binding affinity of −7.1, which lies within the threshold criterion. In molecular interactions, hydrophobic interactions occur between nonpolar molecules in a hydrophilic (water-based) environment. HIS207, ILE203, and ARG102 depict hydrophobic interactions with Cannabidiolic acid in Figure 3.

Hydrogen bonding plays a critical role in enhancing the interaction with the receptor’s active sites. Hydrogen bonding was detected between CBD and polar uncharged residues ASN121, SER205, and positively charged residue ARG190. Residues TRP104, LEU226, PHE192, and VAL126 were observed to participate in alkyl and pi–alkyl interactions with CBD. Additionally, VAL227, ARG102, HIS207, and PHE194 form hydrophobic interactions with the SARS-CoV-2 PLpro receptor (PDB ID: 6W9C), with a binding energy of −7.0 kcal/mol.

In the CBG ligand, five hydrogen bonding interactions were found with the following residues: ALA228, ASP290, ASP294, SER297, and LYS300. Aromatic residue PHE59 was found to form a pi–alkyl interaction with the target protein. Additionally, hydrophobic interactions were detected between VAL289, Leu296 residues, and the 6W9C receptor, with a binding energy of −5.4 kcal/mol.

### 3.4. Significance of Docking Results and Implications for Therapeutic Development

#### 3.4.1. Comparison with Existing Drug Candidates

**Figure 4:**
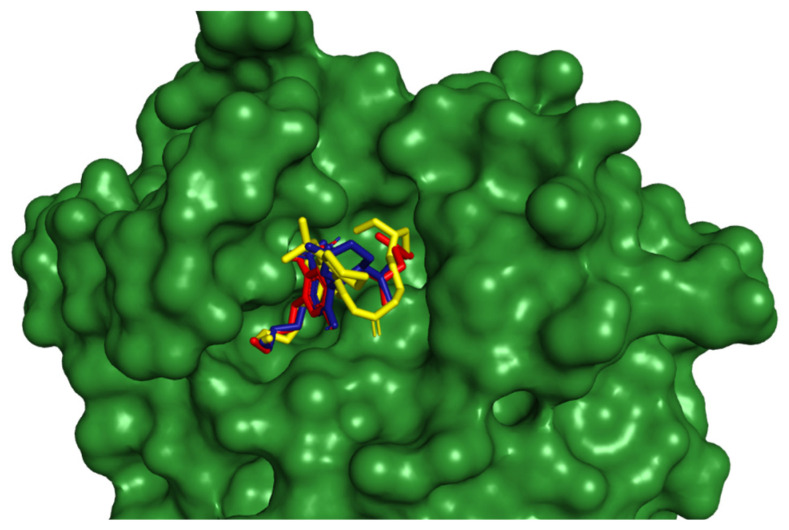
Cannabinoid superimposition with existing drug candidate.

The docking results were compared with existing drug candidates, including remdesivir and lopinavir, to assess the potential of cannabinoid compounds for therapeutic development. When compared to known drug candidates, our ligands demonstrated competitive binding affinities for the PLpro protein. The computed binding energies revealed robust interactions with the protein, with our ligands exhibiting higher binding affinities than existing drugs. Some ligand poses explored a wide range of chemical space, focusing on allosteric pockets and surface residues that previous therapeutic candidates could not address. This investigation into alternative binding locations may provide potential for generating therapeutically useful drugs with novel modes of action and lower resistance. Figure 4 depicts the superimposition of cannabinoids with existing drug candidates.

#### 3.4.2. Statistical Analysis and Data Presentation

**Figure 5:**
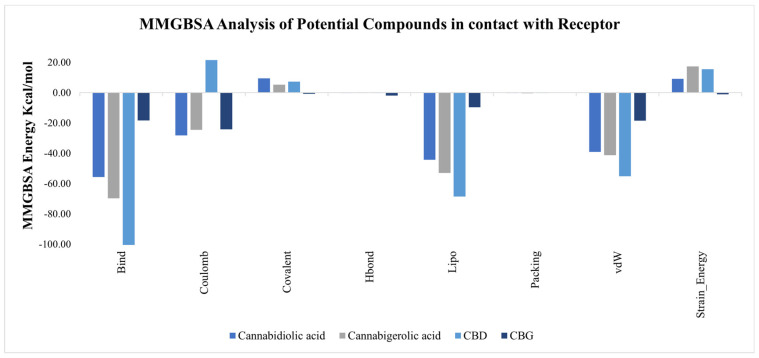
Binding free energy calculations via MMGBSA analysis.

The binding energy of ligands to protein molecules is commonly assessed using the MMGBSA method [70]. The binding free energies of all complexes and the impact of non-bonded interaction energies were assessed. The binding energy of the Cannabidiolic acid complex was −55.73 kcal/mol, while the binding free energy of the CBGA complex was −69.68 kcal/mol. Similarly, CBD and CBG showed binding free energy values of −101.448 kcal/mol and −18.313 kcal/mol, respectively. Gbind is governed by non-bonded interactions such as Gbind–Coulomb, Gbind–Packing, Gbind–Hbond, Gbind–Lipo, and Gbind–vdW (Table 4). Across all interaction types, the Gbind–vdW, Gbind–Lipo, and Gbind–Coulomb energies had the largest effects on the average binding energy. The Gbind–Covalent and Gbind–StrainEnergy energies depict mild contributions. Conversely, Gbind–Packing contributed the least to the final average binding energies (Figure 5). Furthermore, based on their Gbind–Hbond interaction values, protein complexes demonstrated stable hydrogen bonds with amino acid residues. CBD possesses higher negative values compared to others and depicts stronger interaction with the targeted receptor. Consequently, the calculations provided strong support for the binding energy derived from the docking data [70].

### 3.5. MD Simulations of PLpro–Cannabinoid Compounds

#### 3.5.1. Structure Stability Using Root Mean Square Deviation (RMSD)

**Figure 6:**
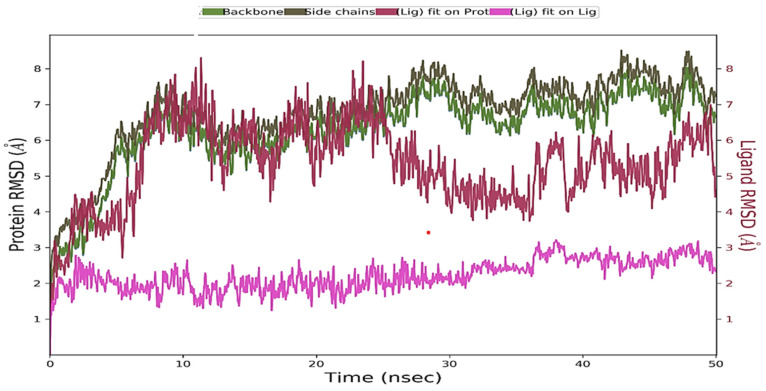
Protein and ligand RMSD plot.

RMSD is a primary measure of the structural changes occurring during the course of simulation (Figure 6). The backbone and side chains of the protein seem to have equilibrated as their RMSD values are not fluctuating drastically towards the end of the simulations. Similar to the protein, the ligand too seems to have equilibrated, given minimal change in its RMSD. The fluctuating RMSD of the ligand with respect to the protein ((Lig) fit on Prot) in the initial time frames indicates the steric adjustment of the ligand to the protein; however, the RMSD seems to be stabilized towards the end of the simulation.

The RMSD summarizes the overall structural changes occurring within the molecule of interest. This statistic is a function of the total change occurring in the positions of the atoms throughout the simulation, with their initial position before the start of the simulation as a reference. An RMSD value per molecule is therefore a sum of all atomic displacements within the molecule during the simulations, compared to their initial positions.

The results shown in Figure 6 indicate the following:

- The overall structural stability of the protein fluctuates; however, it seems to have reached equilibrium towards the end of the simulation. The overall changes in the RMSD range between 5 Å to 8 Å, with fluctuations of 3 Å or less observed from 10 ns to 50 ns (backbone and side chains).
- The overall structural stability of the ligand does not undergo major fluctuations, indicating that the ligand has equilibrated (Lig fit lig).
- When bound to the protein, the RMSD of the ligand fluctuates in the initial stages but eventually reaches equilibrium. Given that the RMSD of the bound ligand is not significantly higher than that of the protein, it seems that, by the end of the simulations, the ligand is still bound in the initial binding pocket (Lig fit pot).

#### 3.5.2. Root Mean Square Fluctuation (RMSF)

RMSF captures signals similar to RMSD at the level of individual residues (amino acids). The RMSF metric highlights fluctuations occurring at the level of residues with respect to the α-Carbon atom and the backbone atoms. Vertical light green lines indicate residues that are interacting with the ligand. Overall, residues interacting with the ligand are concentrated in regions with low fluctuations (<2.4 Å).

Similarly, in capturing residue-specific fluctuations for the protein, this page also provides information on atom-specific fluctuations occurring within the ligand molecule throughout the simulation. Fluctuations in ligand atoms are larger when bound to the protein (Fit Ligand on Protein) compared to the unbound state (Ligand). These fluctuations are expected, as they may be introduced by protein–ligand interactions.

#### 3.5.3. Protein Secondary Structure Analysis

Continuing from the RMSF results, this section summarizes the structural makeup of the protein over the course of the simulation. The figure on Page 5 illustrates the overall composition of secondary structural elements (SSEs) (*X*-axis—residue number, *Y*-axis—participation of the residue in forming secondary structure elements). SSEs primarily consist of alpha helices and beta-sheets/strands. In total, approximately 44% of the protein sequence participates in forming SSEs.

The figure at the top of page 6 summarizes the percentage of SSEs in the protein structure throughout the simulation (*X*-axis—time (in ns), *Y*-axis—% of SSEs). This proportion remains consistently close to the initial 44% throughout the simulation. The figure at the bottom of page 6 tracks the residue-specific contribution to the formation of SSEs throughout the simulation (*X*-axis—time (in ns), *Y*-axis—residues).

#### 3.5.4. Protein–Ligand Contacts throughout the Entire Simulation

**Figure 7:**
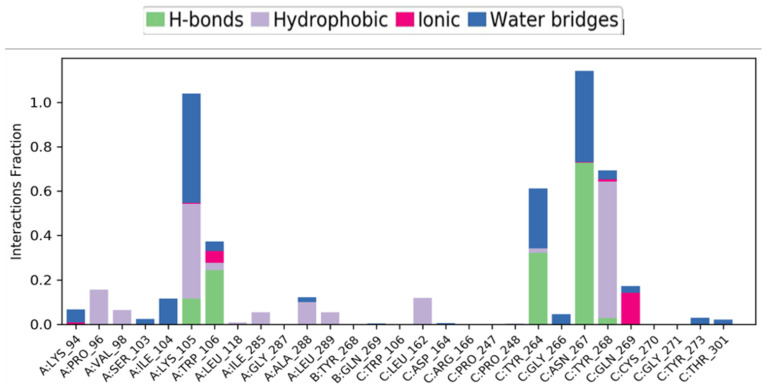
Protein residues interacting with the ligand CBGA.

From Figure 7, it is observed that residues ASN 267, TYR 268, TYR 264, LYS 105, and TRP 106 have multiple interactions with the ligand molecule, among which LYS 105, TYR 264, and ASN 267 form a higher number of water-bridge bonds. Additionally, TYR 264, ASN 267, and TRP 106 form a higher number of hydrogen bonds. This can be further observed in Figure 2B, where ASN 267 consistently forms protein–ligand contacts throughout the simulation. LYS 105, TYR 264, and TYR 268 also interact with the ligand for a significant portion of the simulation time.

Note: (ASN: Asparagine, TYR—Tyrosine, LYS—Lysine, and TRP—Tryptophan. These are standard three-letter notations for amino acids (find them here—https://www.cup.uni-muenchen.de/ch/compchem/tink/as.html accessed on 16 September 2023).

In the context of protein residues interacting with a ligand, the letters A, B, and C typically refer to specific amino acid residues within the protein structure based on the following breakdown:

Amino Acid Residues (A, B, C):

Proteins consists of chains of amino acids. Each amino acid is represented by a single-letter code (e.g., A for alanine, B for asparagine, C for cysteine, etc.). When gaining an understanding of protein–ligand interactions, researchers generally focus on specific amino acid residues within the protein’s binding pocket or active site. These residues play a critical role in determining how the protein interacts with ligands (small molecules, ions, or other proteins).

Binding Pockets and Interactions:

The binding pocket is a specific area of the protein where ligands bind. Amino acid residues within this pocket form interactions with the ligand, influencing the binding affinity and specificity. These interactions can involve hydrogen bonds, Van der Waals forces, electrostatic interactions, and hydrophobic interactions.

Labeling Residues:

Residues are labeled based on their position in the protein sequence. For example, A might represent the first amino acid residue in the binding pocket, B the second, and so forth. Researchers use these labels to identify and analyze specific interactions between the ligand and individual residues. In summary, A, B, and C refer to specific amino acid residues within a protein’s binding pocket, and their interactions with ligands are crucial for understanding protein function and drug design [71,72].

Figure 7 displays protein amino acids on the *X*-axis (refer to the link above to convert the three-letter notation to their amino acid names), with the *Y*-axis quantifying their interaction time with CBGA (the higher the bar plot, the longer the interaction time). If you refer to the docking report, you will notice that some residues noted in the graph overlap with the docking results. Specifically, LYS105, TRP106, TYR264, and TYR268 were shown to interact both in the docking and simulation experiments. However, ASN267 also emerges as an additional amino acid that interacts with CBGA during the simulations.

The potential protein–ligand contact points are characterized by their nature. The residues making contact with the ligand molecule are listed on the *X*-axis, with the *Y*-axis quantifying the time of interactions in terms of the total simulation time. The bar plot is colored based on the types of interaction each residue had with the ligand molecule. From the plot, it can be observed that the top three residues in terms of interaction time with the ligand were ASN267, LYS105, and TYR268. Most bonds formed in these interactions were via water bridges, which are hydrogen bonds formed via water molecules. This schematic represents residue and ligand interactions that lasted for more than 30% of the total simulation time.

#### 3.5.5. Time Course of Interacting Residues and Ligands

**Figure 8:**
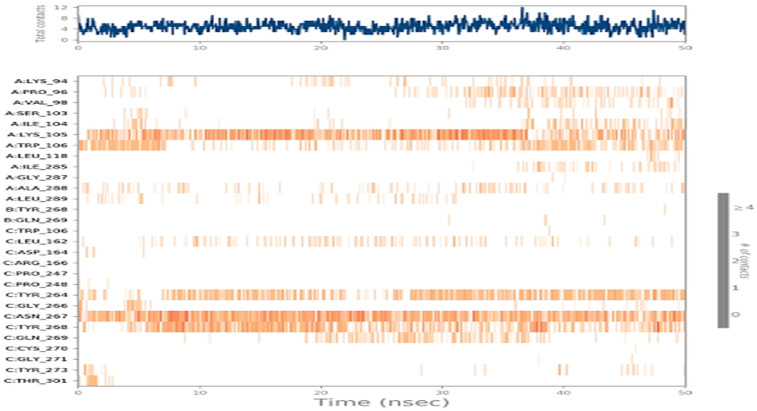
Protein residues interacting with the ligand molecule across the time course.

Figure 8 captures similar information but only like a heatmap plot. In the upper plot of Figure 8, the *X*-axis indicates the simulation time (50 nanoseconds), and the *Y*-axis shows the number of bonds formed per timeframe. This plot captures the total number of bonds formed between protein-CBGA through simulations. In the lower plots of Figure 8, the *X*-axis indicates the simulation time (50 nanoseconds), and *Y* axis contains various amino acids that interacted with CBGA during the course of the simulation. The top plot shows the total number of protein and ligand contacts across the simulation time of 50 nanoseconds (*X*-axis—simulation time (in ns) and *Y*-axis—number of contacts). The bottom plot highlights the residue-specific number of contacts formed with the ligand molecule. Here, darker colors indicate more contacts (*X*-axis—simulation time (nanoseconds) and *Y*-axis—residues). Here, darker colors indicate more interactions.

#### 3.5.6. Ligand Torsion Profile

These plots provide a summary of the conformational changes occurring in every rotatable bond within the ligand throughout the simulation. The ligand torsion profile captures various properties of the ligand throughout the simulations.

For example, the first plot at the top displays the ligand-specific RMSD. Each rotatable bond is represented by a dial and a bar plot. The dial plot summarizes the torsion conformation throughout the simulation, while the bar plot presents the corresponding potential of the rotatable bond on the *Y*-axis.

## 4. Discussion

### 4.1. Interpretation of Docking Results in the Context of PLpro Inhibition

Docking simulations have uncovered crucial insights into the molecular interactions between cannabinoid compounds and SARS-CoV-2’s PLpro protein. The data revealed that cannabinoid compounds exhibit high binding affinities for PLpro, comparable to known drug candidates, suggesting their potential as effective inhibitors of PLpro activity—a critical step in the viral life cycle. Some ligand poses explored alternative binding sites, offering potential for developing novel drugs with unique modes of action and reduced resistance.

These docking studies provide structural insights into how cannabis drugs impact PLpro function by targeting its active site or allosteric regions. This interference could disrupt essential protein–protein interactions or enzymatic activity necessary for viral replication, immune evasion, and host cell manipulation. Additionally, the selectivity of cannabis compounds for PLpro highlights their potential as selective antiviral agents with minimal off-target effects.

The discovery of high-affinity cannabinoid drugs targeting PLpro, such as Cannabidiolic acid and CBGA, holds promise for developing antiviral therapies against SARS-CoV-2. Inhibiting PLpro activity may impede viral replication, reduce viral load, and attenuate viral pathogenicity, potentially slowing disease progression and transmission. Furthermore, targeting PLpro could complement existing antiviral strategies, leading to improved therapeutic outcomes.

### 4.2. Implication of Understanding PLpro Inhibition Mechanisms

PLpro plays a multifaceted role in viral replication, immune evasion, and host cell manipulation, making it an attractive target for antiviral therapy. Identifying specific residues and structural features involved in PLpro inhibition can help tailor therapeutic strategies to selectively disrupt vital protein–protein interactions or enzymatic activity crucial for viral pathogenicity. Targeted antiviral approaches inhibiting PLpro hold promise for broad-spectrum effectiveness against various coronaviruses and other viral illnesses.

Understanding pathways of PLpro inhibition is pivotal for overcoming drug resistance, a significant hurdle in drug development. Targeting conserved regions of PLpro or utilizing allosteric binding sites can yield inhibitors less susceptible to resistance mutations and with prolonged efficacy against emerging viral strains. Additionally, combination therapy targeting multiple phases of the viral life cycle, including PLpro inhibition, may mitigate drug resistance while enhancing treatment outcomes. Insights into how PLpro influences host–pathogen interactions provide valuable understanding of COVID-19 pathogenesis and SARS-CoV-2 evasion mechanisms from the host immune system.

### 4.3. Comparison of Binding Affinities and Interaction Patterns among Cannabinoids

The study examined the binding affinities of four cannabinoid compounds towards specific receptors, revealing significant variations in their interactions. Some cannabinoids exhibited strong binding to PLpro, while others showed lower affinity. These differences likely stem from variations in their chemical structures and functional groups.

Analysis of cannabinoid–PLpro interactions uncovered diverse binding mechanisms and chemical interactions. Certain compounds formed hydrogen bonds, alkyl interactions, and hydrophobic contacts with key receptor residues, while others relied on hydrophobic and π-interactions. The orientation and structural flexibility of cannabinoids also influenced their interactions and binding affinities.

Structural features like aromatic rings, hydroxyl groups, and alkyl chains played crucial roles in determining cannabinoid–receptor interactions. Compounds with aromatic rings and hydrophobic moieties formed strong interactions with PLpro, while alterations in chemical structure led to changes in binding patterns and affinities. Cannabinoids with high receptor affinity may offer therapeutic benefits like inflammation modulation and neuroprotection without adverse effects.

### 4.4. Potential Therapeutic Applications and Drug Development Strategies

Further exploration of the therapeutic potential of the studied chemicals is essential to optimize them for clinical applications. Cannabinoid structures can be enhanced to improve pharmacological properties like potency, selectivity, and metabolic stability through structure–activity relationship (SAR) studies and medicinal chemistry techniques. Lead compounds must undergo thorough preclinical evaluation to assess efficacy, safety, and pharmacokinetics, including animal studies, toxicity profiling, and dose optimization. Promising candidates can advance to clinical trials (Phases I, II, and III) to evaluate safety, efficacy, and tolerability in humans. Additionally, assessing off-target effects, bioavailability, formulation, regulatory approval, and market access are vital steps in the drug development process.

### 4.5. Potential Mechanisms of Action of Cannabinoids on SARS-CoV-2 PLpro

Cannabinoids may have an antiviral effect on SARS-CoV-2 by decreasing the enzymatic activity of PLpro, a major viral non-structural enzyme involved in viral replication and immune evasion. PLpro is an important target for antiviral treatments because it cleaves viral polyproteins and inhibits host immune responses. Cannabinoids may reduce PLpro enzymatic activity by binding to its catalytic site or allosteric regions, affecting viral polyprotein processing and preventing viral replication.

### 4.6. Limitations of the Study and Future Directions

In silico methods accurately predict drug–receptor interactions, but experimental validation is crucial for confirming binding affinities and biological activities. However, limited resources and time constraints may hinder comprehensive validation studies, leading to uncertainty in predictions. To address this, we propose expanding the project into wet lab experiments. Specifically, guided by the computational analysis and molecular dynamics simulations conducted in our study exploring cannabinoids as potential inhibitors of SARS-CoV-2 papain-like protease, we could undertake site-directed mutagenesis to design mutants of the protease and replace key amino acids identified from in silico studies. For instance, we could introduce oppositely charged amino acids at crucial binding sites and assess their impact on binding affinity using techniques such as isothermal titration calorimetry (ITC) or surface plasmon resonance (SPR) assays. Quantifying changes in binding affinity through these experiments can provide valuable insights into the molecular interactions. Given the relatively short duration (50 nanoseconds) of the MD simulations conducted in our study, it is essential to acknowledge potential limitations in capturing long-timescale dynamics and to interpret the results with caution. Additionally, we could explore off-target effects by performing competitive binding assays with related proteins. Furthermore, conducting preclinical and clinical validation studies for safety, efficacy, and pharmacokinetics of lead compounds can be pursued based on the findings from these wet lab experiments.

## 5. Conclusions

In the current study, we employed in silico drug discovery methodologies to identify possible cannabinoid compounds that target PLpro. Structure-based molecular docking and molecular dynamics simulations thoroughly assessed the binding affinities and interactions of potential cannabinoids with the target protein. The study has provided valuable insights into the potential of cannabinoid compounds as effective inhibitors of SARS-CoV-2 PLpro activity. The results demonstrate high binding affinities of cannabinoids, particularly Cannabidiolic acid (CBDA) and Cannabigerolic acid (CBGA), for PLpro, comparable to existing drug candidates. These findings suggest promising avenues for the development of novel antiviral therapies against COVID-19. The detailed analysis of cannabinoid–PLpro interactions has revealed diverse binding mechanisms and interaction patterns, influenced by the structural features of cannabinoids. Compounds with aromatic rings, hydroxyl groups, and alkyl chains exhibited strong interactions with key receptor residues, highlighting the importance of specific chemical moieties in determining binding affinity.

The active site of SARS-CoV PLpro, characterized by a conventional catalytic triad composed of Cys112–His273–Asp287, represents a pivotal locus for enzymatic function. Our study has revealed intriguing insights into the dynamics of ligand binding, albeit without direct proximity to the catalytic center. Despite the absence of immediate interaction with the active site, the binding of these molecules induces notable structural fluctuations within the enzyme. Such alterations in conformational dynamics hold the potential to exert consequential effects on the functional integrity of PLpro.

Understanding the pathways of PLpro inhibition is crucial for overcoming drug resistance and optimizing therapeutic strategies. Targeting conserved regions of PLpro or utilizing allosteric binding sites may yield inhibitors with prolonged efficacy against emerging viral strains. Combination therapy targeting multiple phases of the viral life cycle, including PLpro inhibition, holds promise for mitigating drug resistance and enhancing treatment outcomes. Further exploration of cannabinoid compounds is warranted to optimize their pharmacological properties through structure–activity relationship studies and medicinal chemistry techniques. Preclinical evaluation, followed by clinical trials, will be essential for assessing efficacy, safety, and tolerability in humans. Additionally, investigating the potential mechanisms of action of cannabinoids on SARS-CoV-2 PLpro could provide valuable insights into their antiviral effects and contribute to the development of effective therapeutic interventions against COVID-19.

In summary, targeting PLpro with cannabinoids offers a promising approach for inhibiting viral replication, attenuating pathogenicity, and enhancing therapeutic outcomes in the fight against COVID-19 and other viral infections. Future research efforts should focus on translating these findings into clinically viable treatments to address the ongoing global health crisis.

## Figures and Tables

**Table 1:** Physicochemical properties of selected compounds.

	CBG	Cannabidiolic Acid	CBD	Cannabigerolic Acid
MW (g/mol)	173.17	358.5	511.79	360.5
HBD	3	3	0	3
HBA	5	4	4	4
RBs	3	7	14	10
XLogP	−3.1	6.6	8.9	7.5
Heavy Atom	12	26	37	26
TPSA (Å^2^)	101	77.8	38.8	77.8
Molecular Structure	C_21_H_32_O_2_	C_22_H_30_O_4_	C_21_H_30_O_2_	C_22_H_32_O_4_

**Table 2:** Summary of in silico docking interactions of cannabinoids and SARS-CoV-2 PLpro protein target.

Serial No.	Compound Name	Binding Affinity (kcal/Mol)	Interacting Residues	Type of Bonds
1	Cannabidiolic acid (CBDA)	−5.3	PRO248	Pi–Alkyl: 2 Conventional Hydrogen Bond: 1
			TYR264	
			GLY266	
2	Cannabigerolic acid (CBGA)	−6.1	LYS105	Alkyl and Pi–Alkyl: 8
			TRP106	
			TYR264	
			TYR268	
			ALA288	
			LEU289	
3	CBD	−5.0	TYR268	Alkyl: 3 Pi–Pi T-shaped: 1
			ALA288	
			LEU289	
4	CBG	−4.6	LYS105	Alkyl: 3 Conventional Hydrogen Bond: 4
			TRP106	
			ASN267	
			ALA288	
			LEU289	

**Table 3:** Binding affinities of selected compounds and SARS-CoV-2 PLpro protein along the interacting residues and type of interactions.

Compounds	Binding Affinities (kcal/mol)	Interacting Residues	Type of Interactions	Van der Wall’s Interacting Residues
Cannabidiolic acid	−7.1	VAL126, PHE192, LEU226, VAL227	Pi–sigma, pi–pi T-shaped, alkyl, pi–alkyl	VAL120, ASN121, ARG102, GLY103, ILE119, TRP104, ILE128, HIS207, ILE203, ARG190
Cannabigerolic acid	−7.2	VAL126, PHE192, LEU226, ILE101, TRP104, VAL227	Pi–sigma, pi–pi T-shaped, alkyl (2), pi–alkyl (2)	ARG190, HIS207, PHE92, SER94, ARG102, ASN121, VAL120, VAL127, GLY103, ILE119, TYR170, ILE128, ILE203
CBD	−7	ASN121, SER205, ARG190, VAL126, TRP104, LEU226	H-bond (3), alkyl (2), pi–alkyl (2)	THR124, ASN125, ILE119, ILE203, VAL227, ILE128, PHE194, ILE101, GLU96, ARG102, HIS207
CBG	−5.4	LYS300, SER297, ASP294, ASP290, ALA288, PHE59, VAL289	H-bond (5), pi–alkyl, carbon hydrogen bond	PHE58, LEU296

**Table 4:** The MMGBSA binding free energy calculations of the complexes.

Components	Cannabidiolic Acid (kcal/mol)	Cannabigerolic Acid (kcal/mol)	CBD (kcal/mol)	CBG (kcal/mol)
ΔG_bind_	−55.73	−69.69	−101.45	−18.31
ΔG_bind_-Coulomb	−28.22	−24.51	21.44	−24.18
ΔG_bind_-Covalent	9.42	5.27	7.37	−0.70
ΔG_bind_-Hbond	−0.28	−0.30	−0.25	−1.93
ΔG_bind_-Lipo	−44.26	−52.89	−68.59	−9.70
ΔG_bind_-Packing	−0.31	−0.42	−0.10	0.00
ΔG_bind_-vdW	−39.05	−41.19	−55.13	−18.51
ΔG_bind_-StrainEnergy	9.06	17.29	15.57	−1.05

## Data Availability

The original contributions presented in the study are included in the article, further inquiries can be directed to the corresponding authors.
